# Correlations between social-emotional feelings and anterior insula activity are independent from visceral states but influenced by culture

**DOI:** 10.3389/fnhum.2014.00728

**Published:** 2014-09-16

**Authors:** Mary Helen Immordino-Yang, Xiao-Fei Yang, Hanna Damasio

**Affiliations:** ^1^Brain and Creativity Institute, University of Southern CaliforniaLos Angeles, CA, USA; ^2^Rossier School of Education, University of Southern CaliforniaLos Angeles, CA, USA; ^3^Neuroscience Graduate Program, University of Southern CaliforniaLos Angeles, CA, USA; ^4^Dornsife Cognitive Neuroscience Imaging Center, University of Southern CaliforniaLos Angeles, CA, USA

**Keywords:** arousal, subjective affect, cultural neuroscience, interoception, East-West differences

## Abstract

The anterior insula (AI) maps visceral states and is active during emotional experiences, a functional confluence that is central to neurobiological accounts of feelings. Yet, it is unclear how AI activity correlates with feelings during social emotions, and whether this correlation may be influenced by culture, as studies correlating real-time AI activity with visceral states and feelings have focused on Western subjects feeling physical pain or basic disgust. Given psychological evidence that social-emotional feelings are cognitively constructed within cultural frames, we asked Chinese and American participants to report their feeling strength to admiration and compassion-inducing narratives during fMRI with simultaneous electrocardiogram recording. Trial-by-trial, cardiac arousal and feeling strength correlated with ventral and dorsal AI activity bilaterally but predicted different variance, suggesting that interoception and social-emotional feeling construction are concurrent but dissociable AI functions. Further, although the variance that correlated with cardiac arousal did not show cultural effects, the variance that correlated with feelings did. Feeling strength was especially associated with ventral AI activity (the autonomic modulatory sector) in the Chinese group but with dorsal AI activity (the visceral-somatosensory/cognitive sector) in an American group not of Asian descent. This cultural group difference held after controlling for posterior insula (PI) activity and was replicated. A bi-cultural East-Asian American group showed intermediate results. The findings help elucidate how the AI supports feelings and suggest that previous reports that dorsal AI activation reflects feeling strength are culture related. More broadly, the results suggest that the brain's ability to construct conscious experiences of social emotion is less closely tied to visceral processes than neurobiological models predict and at least partly open to cultural influence and learning.

## Introduction

Emotions fundamentally involve body responses (James, 1894), and the neural mapping of these responses is thought to form the basis for emotional experiences, or feelings (Craig, 2002; Damasio and Carvalho, 2013)—i.e., for the subjective, conscious perception that one feels emotionally affected or “moved” by a situation. Like all feelings of body states, such as feelings of cold or heat (Craig et al., 2000), hunger or fullness (Del Parigi et al., 2002), feelings of emotion-related physiological states are thought to come most fully into awareness in the anterior insula (AI). Though various cortical and subcortical systems contribute to emotion, mood, and their regulation (e.g., amygdala, hypothalamus, cingulate cortex), the AI is the cortical terminus for the interoceptive maps from which conscious affective experiences are thought to be constructed, such as maps of emotion-related heart-rate changes (Craig, 2002; Critchley et al., 2005).

In keeping with theoretical accounts, the AI is activated when individuals experience emotions (Damasio et al., 2000; Lamm and Singer, 2010) and recent studies have demonstrated that social-emotional feelings activate AI regions that overlap with regions activated during interoceptive awareness (e.g., Zaki et al., 2012). However, correlation of the AI's real-time activity with visceral responses and emotional feelings has focused on disgust reactions to viewing body violations (e.g., Harrison et al., 2010) or on affective experiences of body stimulation as noxious or pleasant (e.g., Craig et al., 2000). Although the AI is activated during emotions that rely on complex reasoning about the social context, e.g., admiration for virtue and compassion (Immordino-Yang et al., 2009), it is not clear how the activations correlate with visceral responses and with feeling strength in these emotions.

This question is especially pertinent given psychological evidence that constructing cognitively complex feelings is a dynamic, socially mediated, inferential process (Barrett et al., 2007; Immordino-Yang, 2010; Seth, 2013) that is shaped by childhood and adolescent social experience (Eisenberg et al., 2006). Reactions such as those to another's virtue or social pain require the onlooker to understand the broader circumstances of the other person's actions and from there to make social-cognitive inferences about that person's subjective experiences and qualities of mind. The onlooker's resulting physiological reaction (the emotion) and experience (the feeling; Damasio, 1999) cannot rely entirely on empathy as he or she experiences a different emotion than the other person shows. (For example, an onlooker may experience compassion when watching a man dine alone because he knows that the man's wife recently died. Or, the onlooker may feel admiration for the virtue of a medical doctor who sacrifices her own safety, affluence and comfort to work in a remote region with dangerous health conditions—the doctor appears “concerned and caring” but the onlooker feels “inspired.”) Because social-emotional feelings are so heavily dependent on interpretation and inference, it is possible that they may not be as tightly tied to visceral reactions as are other feelings. It is also possible that individuals construct social-emotional feelings in culturally variable ways (Barrett, 2012; Immordino-Yang, 2013), and that neural activity may therefore correlate with feelings differently across cultural groups. These issues have not been investigated, despite their implications for neurobiological and psychological models of emotional feelings and sociality.

Given the above, we investigated how AI activity correlates in real-time with cardiac arousal (an index of interoception), and with social-emotional feeling strength (an index of a psychological process). During simultaneous fMRI and electrocardiogram (ECG) recording, participants reported trial-by-trial how strongly emotional they felt to each of a series of short video narrative stimuli piloted to induce social emotions of varying strengths. We then analyzed AI activations during strong and weak emotional experiences, and the trial-by-trial correlations between activity magnitude in the AI, cardiac arousal and feeling strength. We focused the psychological measure in our study on social-emotional feeling strength because intensity of subjective affect is a centrally important and ubiquitous dimension of emotional experience.

Because we were also interested to test whether cultural background influences the neural processing of conscious emotional feelings, we included Chinese participants recruited in Beijing (CH) and two groups of Americans recruited in Los Angeles: one mixed-ethnicity group of monolingual English-speakers not of Asian descent (representative American group, RA) and 2nd generation East-Asian group (Asian American, AA). Emotion-related values and norms are different between China and the United States (e.g., Markus and Kitayama, 1991; Tsai, 2007). We examined each group's data separately, and then statistically compared the three group results. Finding a significant CH to RA group difference would be suggestive of a cultural effect, and finding a hybrid pattern in the AA group, who are genetically Chinese but were raised in a bicultural Chinese-American context, would strengthen the interpretation that the finding is related to developmental cultural exposure.

We separately examined the activity in the ventral and dorsal AI sectors, as they are cytoarchitecturally and functionally distinct (Mesulam and Mufson, 1982a; Kurth et al., 2010), and there are open questions as to how each supports feelings (Lamm and Singer, 2010). The ventral AI is an evolutionarily old sector (Mesulam and Mufson, 1982a) involved in autonomic modulation (Mesulam and Mufson, 1982b; Mutschler et al., 2009) and consistently implicated in fMRI studies of emotion processing (Kurth et al., 2010). The dorsal AI is evolutionarily newer (Mesulam and Mufson, 1982a) and more visceral somatosensory related (Mesulam and Mufson, 1982b; Craig, 2002). The dAI has been implicated in aspects of cognition/decision-making (Mutschler et al., 2009; Kurth et al., 2010) and in interoceptive and emotional awareness (Critchley et al., 2004; Zaki et al., 2012). These sectors also display distinct patterns of functional connectivity (Nelson et al., 2010; Deen et al., 2011), and studies have linked individual differences in these sectors' network connectivity to emotional and cognitive functioning (Seeley et al., 2007; Touroutoglou et al., 2012).

In each cultural group, we expected both AI sectors to activate when participants reported feeling emotional, and we expected the activity to correlate with trial-by-trial changes in cardiac arousal. Given these expectations were confirmed, we tested the hypothesis that, for each group, the activity in each AI sector in each hemisphere would correlate with participants' real-time reports of feeling strength, and that, trial-by-trial, the variance in BOLD signal associated with feeling strength would be dissociable from that associated with cardiac arousal (i.e., that the correlation between feeling strength and BOLD signal would hold after controlling for cardiac arousal). Independent variance would be evidence for psychological processing of social-emotional feelings in the AI above and beyond interoception.

We then tested for cultural effects on the BOLD variance correlated uniquely with feelings. That is, we were interested to uncover a cultural group difference in the *correspondence between* fluctuations in BOLD signal magnitude and fluctuations in feeling strength, especially if we would not find an *absolute* difference between the groups in magnitude of BOLD signal change or in magnitude of feelings. A cultural group difference in the correspondence between BOLD signal change and feeling strength would be evidence that developmental exposure to culture influences the process by which individuals construct conscious experiences of social emotions.

Given the novelty of our study, we did not attempt to hypothesize a priori the direction of cultural differences. Instead, we considered that our hypothesis of a cultural effect would be confirmed if: (1) the correlation between AI BOLD signal magnitude and feeling strength showed a significant interaction with cultural group; (2) the results from the AA group were intermediate between the results of the CH and RA groups; and, (3) we could replicate the cultural group difference in a different cohort of CH and RA participants with a different corpus of stimuli.

We also tested for cultural effects on the variance correlated uniquely with cardiac arousal; here we expected a negative result. Interoceptive mapping of cardiac arousal is a basic physiological function, unlike the psychological processing that undergirds emotional feelings (or even interoceptive awareness). We therefore did not expect it to be influenced by social norms.

Finally, as the AI is thought to re-map visceral states previously mapped in the posterior insula (PI; Craig, 2002), we analyzed PI correlations to cardiac arousal and feeling strength, and then controlled for activity in the PI and reanalyzed correlations between AI activity, cardiac arousal, and feeling strength. We expected the PI to be activated to our stimuli. We expected that PI activity would correlate with cardiac arousal but not with feeling strength, as subjective interpretations or experiences of physiological states (feelings) are thought to come most fully into awareness only at the level of the AI. Finally, we hypothesized that controlling for PI activity would render AI correlations to cardiac arousal non-significant but would not alter patterns of correlation between AI activity and feelings. Were these expectations confirmed, it would provide additional evidence that social-emotional feelings are processed relatively independently from body states in the insula.

## Materials and methods

### Participants

Participants were healthy, right-handed, and neurologically normal. [Handedness was assessed using a modified version of the Edinburgh handedness questionnaire (Oldfield, 1971), which ranges from −14 (strongly left-handed) to +14 (strongly right-handed). All participants scored 11 or higher.] Participants ranged in age from 18 to 30 years. All participants reported normal or corrected-to-normal vision and normal hearing. None had a history of neurological or psychiatric disorder, physical or emotional abuse, and none were using psychotropic medication. None reported a medical condition that would preclude scanning. All participants gave written informed consent in accordance with the requirements of the Institutional Review Boards of the University of Southern California (USC) and Beijing Normal University (BNU). Participants were compensated for their participation in accordance with the norms of their university community.

#### Chinese group (CH)

Fifteen monolingual Mandarin-speaking Chinese participants were recruited from the BNU community (7 females; average age 22.9 years, *SD* = 3.47). All were born to monolingual Mandarin-speaking parents and raised in mainland China, and none had resided outside of China.

#### Representative American group, excluding Americans of Asian descent (RA)

Sixteen monolingual English-speaking American participants not of Asian descent (8 females; average age 22.4 years, *SD* = 2.87; 12 Caucasian, 2 Latino, and 2 African-American) were recruited from USC. Participants were raised in the U.S. by monolingual English-speaking American-born parents. The composition of this group was representative of the American student population at USC, except that it did not include participants of Asian descent.

#### East-Asian American group (AA)

Sixteen second-generation East Asian-American participants (8 females; average age 20.4 years, *SD* = 1.44) were recruited from the USC community in Los Angeles. Participants' parents had been born and raised in China (*n* = 14) or Korea (*n* = 2). Participants had lived in the United States from before age 6, the approximate age at which formal schooling begins (13 had been born in the U.S.). All reported English as their primary language.

#### Replication group

Data from additional groups of 14 Chinese and 13 American participants were utilized for the replication study (see below).

### Protocol

Participants were told that they would be exposed to a series of true stories about real people's lives, and that they should feel comfortable reporting their honest feelings in response to each. Following a previously developed protocol (see Immordino-Yang et al., 2009), participants were first introduced to the narrative stimuli in a private preparation session outside of the scanner in which an experimenter verbally recounted the series of scripted narrative stimuli in one of two counterbalanced orders and presented an accompanying full-length video of each protagonist on a laptop computer. Each narrative took approximately 60–90 s total to present, including showing the video (average video length was 43 s). This preparation session was necessary mainly because the narratives in full form were too long to show in the scanner in sufficient numbers to obtain reliable neuroimaging results, and shorter narratives would have been unlikely to induce genuine social emotions beyond empathy (i.e., beyond mirroring of the emotion being displayed by the protagonist). This method also allowed us to systematize the amount of exposure each participant had with each narrative prior to scanning (as compared with leaving participants on their own to familiarize themselves with each narrative). These preparation sessions were also videotaped for future studies of participants' behavior. For more details on the preparation sessions, see Immordino-Yang et al. (under review). The one-on-one preparation session was conducted by an experimenter of the same nationality as the participant (Mary Helen Immordino-Yang conducted the sessions in Los Angeles; Xiao-Fei Yang conducted those in Beijing).

After the preparation session, participants underwent BOLD fMRI with simultaneous electrocardiogram (ECG) recording as they viewed a 5-s segment of each full-length narrative video depicting the crux of the narrative with one sentence of verbal information from the preparation session delivered both auditorily and transcribed underneath the image in stationary text (in Mandarin or English), followed by 13 s of gray screen. For each trial, participants reported via button press the real-time strength of their feeling once they became aware of it (i.e., one button press per trial; this press could happen at any point during the video or gray screen). A cross appeared for 2 s to separate trials. Each narrative was shown twice over the course of the fMRI experiment, never twice during the same run, for a total of 100 trials divided into four runs of approximately 9 min each.

Experimenters stressed to participants that they should report their experienced strength of emotion at the current time (i.e., that they should not report how they remember feeling in the preparation session, or how they imagine the experimenters expect them to feel, or how they might usually feel, etc.). For this purpose, a button box rested in the participant's right hand. Participants could report “no emotion,” “moderate emotion,” “strong emotion,” or “overwhelmingly strong emotion.” Button presses and TR timestamps were collected on an IBM Thinkpad computer (IBM, Armonk, NY) running MATLAB (MATLAB 2007a, student version).

Participants abstained from caffeine, nicotine and medications for 24 h prior to the experiment. (None reported this being unduly challenging.) Each participant arrived at the lab between 9:00 and 9:30 a.m. on the day of their participation. The preparation session ran for 2 h, and scanning began between 12:30 and 1:00 p.m. Following scanning, participants were asked to explain in a second private session how they had felt about each narrative from the experiment while in the scanner, to ensure that the participants had remembered all of the narratives.

### Stimuli

All participants were presented with the same video stimuli, subtitled in the participants' native language. Each stimulus was prepared in two versions. A full length version (average length 43 s) was utilized pre-scan to familiarize participants with the narratives; a 5-s segment from the full video, depicting the crux of the narrative, was shown in the scanner.

Narrative stimuli depicted compelling, true stories featuring real people (not actors) that unfolded like mini documentaries. The corpus of 40 emotional stimuli was balanced for positive and negative stories (stories had been piloted to induce varieties of admiration and of compassion/empathy). Ten additional control stimuli depicted more commonplace, less emotion provoking stories, and had been piloted to elicit equivalent cognitive and social processing as the emotion stimuli, but not to result in strong emotion beyond interest. For consistency, all of the stories aimed to induce affiliative, pro-social reactions; we avoided stimuli that would induce reactions such as moral indignation, hate, contempt, anger, etc. Stimulus categories/properties are as described and justified in Immordino-Yang et al. (2009, Supplemental Information), although the corpus of stimuli was specifically developed for this study and only video stimuli were included.

Stimuli for this experiment were constructed from stories gathered from the Internet, television and other sources in the U.S. and China. Half of the 50 true-life narratives featured protagonists from China (speaking Mandarin) and half featured protagonists from the U.S. (speaking English; none were of Asian descent). (We confirmed that viewing stimuli about protagonists from participants' own vs. the other country produced no effects; see Supplementary Analyses.) Stimuli about Chinese and about American protagonists were matched by content theme. Examples of matched content themes were: “talented physician sacrifices affluent lifestyle and dedicates his/her career to helping the underprivileged” (positive, induces admiration for virtue); “young person displays exceptional musical talent” (positive, induces admiration for skill); “physically disabled person feels lonely and forgotten by previous friends” (negative, induces compassion for social pain); “amateur sports player sustains a painful leg injury” (negative, induces compassion/empathy for physical pain); “professional man becomes a stay-at-home father” (induces control social processing).

Potential stimuli were extensively piloted in Los Angeles and in Beijing for emotional potency and for cultural equivalence (i.e., for the likelihood that the narratives would result in qualitatively similar emotional reactions in Chinese and in American young adults). They were also reviewed for cultural equivalence by an expert in comparative Chinese and American culture at USC and by a Chinese psychologist at BNU. Details on stimulus development and piloting can be provided on request.

### ECG data acquisition and processing

ECG was measured using a BIOPAC MP150 system with three MRI-compatible electrodes placed on the participant's chest and sampled at a rate of 4000 Hz. ECG recordings were preprocessed in AcqKnowledge 9.32 (BIOPAC Systems, Inc.) to extract the R peaks of the QRS complex. The resulting intervals between R peaks were plotted and manually corrected for artifacts. RR interval series were then uniformly re-sampled at 4 Hz (Kubios HRV; kubios.uku.fi/), transformed into heart-rate series (in beat-per-min), and plotted relative to the average heart-rate from the 2-s gray screen preceding the trial (Matlab 2011a). Cardiac arousal was operationalized as the maximum increase in heart-rate across the trial. (We note that peak values fell on average late in the trial, at approximately 10 s post stimulus onset).

### fMRI data acquisition and processing

The same scanner model and sequences were used to collect neuroimaging data at BNU Imaging Center for Brain Research and at USC Dana and David Dornsife Neuroimaging Center. Whole brain images were acquired using a Siemens 3 Tesla MAGNETON TIM Trio scanner with a 12-channel matrix head coil. Functional scans were acquired using a T^*^_2_ weighted Echo Planar (EPI) sequence (*TR* = 2 s, *TE* = 30 ms, flip angle = 90°, acquisition matrix: 64 × 64, *FOV* = 192mm) with a voxel resolution of 3 × 3 × 4.5 mm. We utilized PACE (Prospective Acquisition CorrEction) to automatically correct for motion during data acquisition. Thirty-two continuous transverse slices were acquired to cover the whole brain and brain stem. Functional data were acquired continuously for the duration of each run, with breaks between runs. Anatomical images were acquired using a magnetization prepared rapid acquisition gradient (MPRAGE) sequence (*TI* = 900 ms, *TR* = 1950 ms, *TE* = 2.26 ms, flip angle = 7°) with an isotropic voxel resolution of 1 mm; 160 slices were acquired to cover the whole brain, dimensions: 256 × 256 × 160.

Data from one male Chinese participant were excluded prior to pre-processing due to excessive head movement (the subject coughed repeatedly). (The maximum head motion among the remaining participants was 0.75 mm, three dimensions combined). The remaining data were processed using SPM8 (Wellcome Department of Cognitive Neurology, London, UK) in MATLAB 2009b (MathWorks, Inc.). Functional images were slice timing corrected, aligned to the first volume acquired and co-registered to the anatomical image. Co-registrations were individually examined for each participant in native space to ensure high quality alignment. Then, anatomical images were segmented and non-linearly normalized to MNI space (Montreal Neurological Institute) using the standard probabilistic tissue maps provided in SPM8. To account for size and shape differences between East-Asian and Caucasian brains, affine regularization was set specifically for each group and the results were verified individually for each participant as follows: Individual verifications were conducted first for major landmark structures, lateral boundaries and emotion-related regions for the whole brain, and then for the boundaries and internal structural landmarks within the insular cortex, including for anterior, posterior, dorsal, and ventral insular boundaries and for the principal sulcus of insula. See also below, section Identifying the Volumes of Interest. The same normalization transformation was then applied to the functional images. Finally, the images were smoothed using an 8-mm FWHM Gaussian kernel.

All data were subjected to session-specific grand mean scaling, high-pass filtering with a cut-off period of 128 s and auto-correlation correction using an AR(1) model.

### Sorting the trials by the behavioral data

In certain analyses, we were interested to depict the difference in average BOLD activity level when participants *experienced* genuine emotional feelings (of varying strengths) vs. when they *did not feel* emotional. These analyses are identified throughout the Materials and Methods Section, and included: modeling the task-related BOLD response at the whole-brain level, utilizing functional contrasts to identify the volumes of interest (VOIs), and visualizing the event-related averages (ERAs) for emotion and control in the VOIs. For these analyses, we did not assume that every participant always experienced emotion to the emotion trials and no emotion to the control trials (although this was generally the case; see Table 1). Instead, we included only emotion trials to which participants reported feeling emotional and control trials to which participants reported feeling no emotion, as determined by participants' button press during the trial. We realize that the particular stimuli included in each individual's analysis would differ; however, we reasoned that, given our aim, it would be preferable in these particular analyses to have uniformity of reported feeling strength than uniformity of stimuli (see also Immordino-Yang et al., 2009, Supplementary Information). The number of emotion and control trials included in these analyses did not differ across the groups (emotion: *F*_[2, 43]_ = 1.38, *p* = 0.26, η^2^_*p*_ = 0.060; control: *F*_[2, 43]_ = 0.29, *p* = 0.75, η^2^_*p*_ = 0.013). Excluded trials were modeled as a separate condition of no interest.

**Table 1:** **Behavioral data**.

	**Chinese (CH)**		**East-Asian American (AA)**		**American (RA)**	
	**Emotion**	**Control**	**Emotion**	**Control**	**Emotion**	**Control**
(A) Mean feeling strength *(SD)*	2.27 (*0.35*)	1.38 (*0.25*)	2.26 (*0.34*)	1.40 (*0.30*)	2.22 (*0.44*)	1.30 (*0.21*)
(B) Percentage of successful trials (*SD*)	77.1 (*12.5*)	65.0 (*22.5*)	84.4 (*9.5*)	66.5 (*21.1*)	78.0 (*16.7*)	70.6 (*19.8*)

*(A) Means and standard deviations for button press reports of feeling strength to emotion and control stimuli. Participants could report “1: no emotion,” “2: moderate emotion,” “3: strong emotion,” or “4: overwhelmingly strong emotion.” (B) Percentages of “successful trials,” i.e., emotion trials in which participants reported feeling emotional (button press of 2–4) and control trials in which participants felt no emotion (button press of 1)*.

In all other analyses, all trials were included.

### Modeling the task-related BOLD response at the whole-brain level

In order to capture the complex neural activity during the 18-s trial, each experiment condition (emotion and control) was separately modeled at the individual level using a finite impulse response function (FIR) with 9 time bins, each corresponding to a 2-s TR. For each participant, the parameter estimates for each condition were averaged over the 4th–8th time bins (corresponding to the time window of 6–16 s post stimulus onset) to create contrast maps that captured BOLD signal change relative to the implicit baseline. The time window chosen had previously been shown to capture the BOLD responses in this task (Immordino-Yang et al., 2009), and can be further appreciated by viewing the ERAs in **Figures 3, 5**. (Note: Trials were sorted by behavioral data in this analysis; see above).

### Identifying the volumes of interest (VOIs)

**Figure 1:**
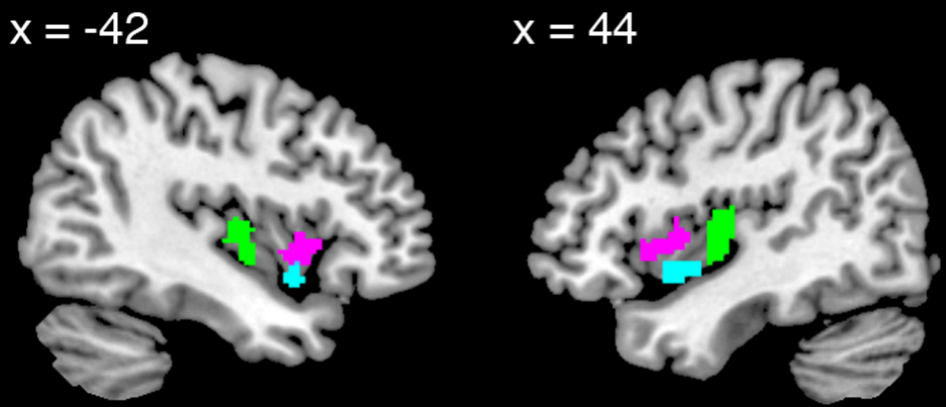
**Views of the dorsal anterior (pink), ventral anterior (turquoise), and posterior (green) insula volumes of interest**.

The contrast maps calculated above for emotion and for control were entered into group-level random-effects full factorial models, one model for the CH group and one for the RA group. The AI was functionally defined from the union of activation clusters from the contrast of emotion vs. control for these two groups, thresholded at *p* < 0.005 uncorrected and displayed on a template brain (provided in MRICron, http://www.nitrc.org/projects/mricron, Colin27 Brain). (The alignment of the insular cortex had previously been confirmed individually for each participant's brain during the preprocessing phase). The posterior boundary was defined dorsally by the principle sulcus, posterior to the third short gyrus (Damasio, 1995), then extended directly downward. (This boundary fell at *y* = −2). The resulting region was divided into dorsal and ventral AI sectors by approximating the cytoarchitectonic boundary described by Mesulam and Mufson (1982a). We then overlaid each VOI on each individual's anatomical image and confirmed that the anatomical localization was accurate for all participants. The VOIs were confirmed by Hanna Damasio, a neuroanatomist (see also Figure 1).

Since the contrast of emotion vs. control yielded only small clusters of activation in the PI, we anatomically defined this VOI using the Automated Anatomical Labeling Atlas (Tzourio-Mazoyer et al., 2002), and defined the anterior boundary at *y* = −4. As above, we overlaid the VOI on each individual's anatomical image and confirmed the accuracy of the anatomical localization.

### Visualizing event-related averages (ERAs) of the VOI BOLD time courses for emotion and control

BOLD time courses from voxels falling within the vAI, dAI, and PI VOIs identified above were averaged to create one time series for each condition, for each VOI, for each individual using MarsBaR (Brett et al., 2002). These were fitted to a finite impulse response function. Resulting beta values corresponding to the average BOLD level for each bin were converted into percent signal change relative to implicit baseline and averaged for each group for each condition and plotted. (Note: Trials were sorted by behavioral data in this analysis; see above.)

### Initial analyses

The aim of the initial analyses was to test whether the stimuli were equally neurally and behaviorally effective in each cultural group, i.e., whether the groups' reports of emotion strength and magnitude of BOLD signal change during emotion differed.

We tested for group differences in the magnitude of BOLD signal change during emotion processing in two ways: First, we used a VOI approach. Working from the ERA plots calculated above, we averaged each group's BOLD magnitude for the emotion condition over the 4th–8th TRs (the time window utilized for modeling the task-related BOLD response at the whole-brain level) and compared the three groups' results using ANOVA. Second, we used a voxel-wise approach. Working from whole-brain contrasts of emotion vs. baseline, we conducted voxel-wise comparisons of BOLD activation across the three groups using ANOVA and examined the results in the AI, PI and other emotion-related and social processing regions (amygdala, anterior cingulate, dorsal and ventral medial and lateral prefrontal cortex, precuneus, and temporal-parietal junction). (Note: Trials had been sorted by behavioral data in these analyses.)

We also tested for effects of viewing in-group vs. out-group stimulus protagonists on the neural and behavioral data, and found none (see Supplementary Analyses).

### Main analyses

#### Examining AI VOI activation levels for emotion vs. baseline for each participant group separately

Working from the ERA plots of emotion calculated above, we averaged each group's BOLD magnitude over the 4th–8th TRs (the time window utilized for modeling the task-related BOLD response at the whole-brain level) and utilized a one-sample *t*-test to determine whether activation was significantly greater than 0.

To confirm that the activations were consistent across positive/rewarding social emotion (i.e., for trials in which stimuli aimed to induce admiration for virtue and for skill) and pain-based/empathic social emotion (i.e., for trials in which stimuli aimed to induce compassion for social pain and for physical injury), we also separately examined the activations associated with stimuli from these sub-categories. We found that both sub-categories of emotion stimuli produced significant activation in all groups in both AI VOIs (see Supplementary Table 1).

#### Examining whole-brain BOLD contrasts of emotion vs. control for each participant group separately

The contrast maps capturing BOLD activation during the emotion and control conditions were entered into group-level random-effects full factorial models, one model for each participant group. We examined the contrast separately for each group, correcting for multiple comparisons using the False Discovery Rate correction at *q*(FDR) < 0.05. (Note: Trials were sorted by behavioral data in this analysis; see above.)

#### Examining trial-by-trial correlations between fluctuations in VOI BOLD responses, cardiac arousal and feeling strength

In these analyses, we aimed to characterize intra-subject co-variation among the measures. To improve the BOLD and ECG signal-to-noise ratios, we first averaged together the data corresponding to the two presentations of the same narrative stimulus (from different runs). That is, for each participant we calculated the average BOLD time-course for each VOI, heart-rate response time-course, and feeling strength (button press value) corresponding to the two presentations of the same narrative. We then identified the peak values for the resulting BOLD and heart-rate response time courses. Peaks were defined as the local maximum of greatest magnitude. Taking into account the hemodynamic delay, we excluded the first two TRs when identifying the BOLD peak. This method produced 50 sets of 3 values for each participant for each VOI. (In the rare case where no peaks were identified within a time course, the corresponding set of values was excluded from further analysis.)

We then calculated for each participant, across the 50 sets of values, for AI VOIs:

1. the partial correlation coefficient between the magnitude of the BOLD peak and the magnitude of the heart-rate peak, controlling for the feeling strength. This calculation was repeated after controlling for PI activity.
2. the partial correlation coefficient between the magnitude of the BOLD peak and the feeling strength, controlling for the magnitude of the heart-rate peak. This calculation was repeated after controlling for the variance shared between the dAI and the vAI VOIs in the magnitude of the BOLD peak and after controlling for PI activity.
  Given the results, we decided *post-hoc* to calculate:
3. the correlation coefficient between the timing of the BOLD peak and the button-press response time (i.e., the time the participant required to become aware of and report the strength of their current emotional feeling).

We tested for effects of cultural group on these correlation coefficients using ANOVA, and utilized 2-tailed, one sample *t*-tests to characterize the consistency of the correlations within each cultural group for each AI VOI.

Finally, we tested correlations between PI activity, cardiac arousal, and feeling strength.

We conducted all tests on the left and right VOIs separately and every result was consistent between the left and right; therefore, results reported are bilateral.

Notes: The main analyses were designed to avoid directly comparing the magnitude of BOLD signal collected from different scanners. The trial-by-trial analyses were designed to additionally avoid the problem of multiple comparisons (and the associated problem of overestimated effect sizes) when statistically comparing the cultural groups. The inclusion of the AA group served in part to verify that cultural differences were not due to anatomical normalization issues with Asian brains.

## Results

### Initial results

**Figure 2:**
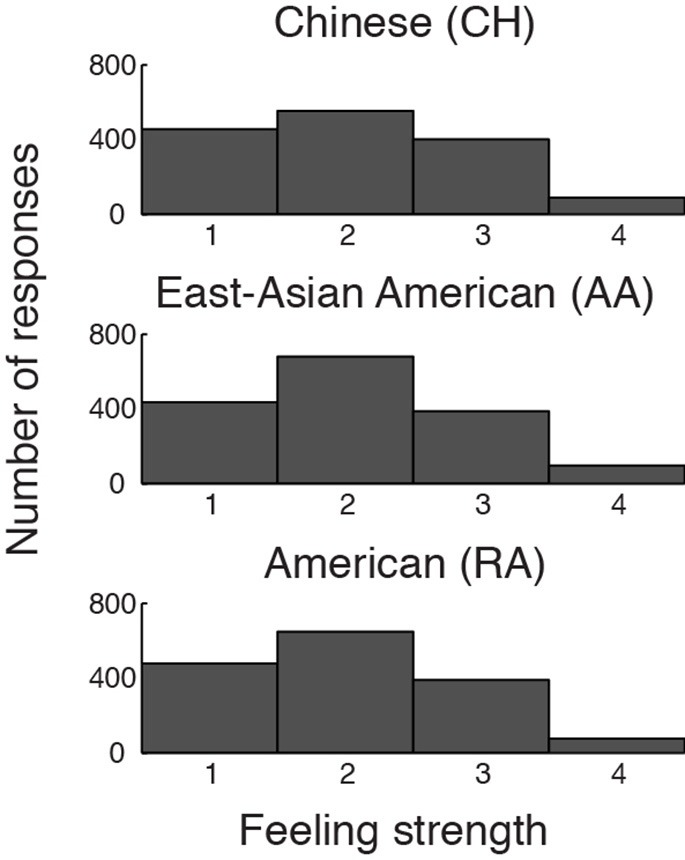
**Distributions of participants' button-press responses during fMRI/ECG, describing the strength of their emotional feelings to each stimulus**. Participants could report “no emotion” (1), “moderate emotion” (2), “strong emotion” (3), or “overwhelmingly strong emotion” (4). The groups' distributions do not differ significantly.

Validating the experiment protocol, the emotion stimuli were equally neurally and behaviorally effective in each cultural group. Every participant reported feeling emotional during the experiment. There were no cultural group differences in participants' reported strength of feelings (*F*_[2, 43]_ = 0.16, *p* = 0.86, η^2^_*p*_ = 0.007) or in the proportion of scanner trials to which participants reported feeling emotional (*F*_[2, 43]_ = 1.38, *p* = 0.27, η^2^_*p*_ = 0.060; see also Figure 2 and Table 1). Utilizing the VOI approach, we found no differences in AI BOLD activation during emotion relative to baseline in either hemisphere (dAI: *F*_[2, 43]_ = 0.55, *p* = 0.58, η^2^_*p*_ = 0.025; vAI: *F*_[2, 43]_ = 1.12, *p* = 0.33, η^2^_*p*_ = 0.050; results reported are bilateral). Utilizing the voxel-wise approach, the peak effect size in the AI was *F*_[2, 43]_ = 2.47, *p* = 0.09 uncorrected (not a significant difference).

We found no significant differences in activation magnitude in any of the other emotion-related or social processing regions examined, even at the lenient threshold of *p* < 0.005 uncorrected for multiple comparisons.

The post-scan interviews revealed that, almost universally, participants remembered every narrative and described pro-social, affiliative reactions; i.e., they reported liking/feeling sympathetic toward/feeling engaged with the protagonists.

### Main results

#### Emotion vs. baseline processing

**Figure 3:**
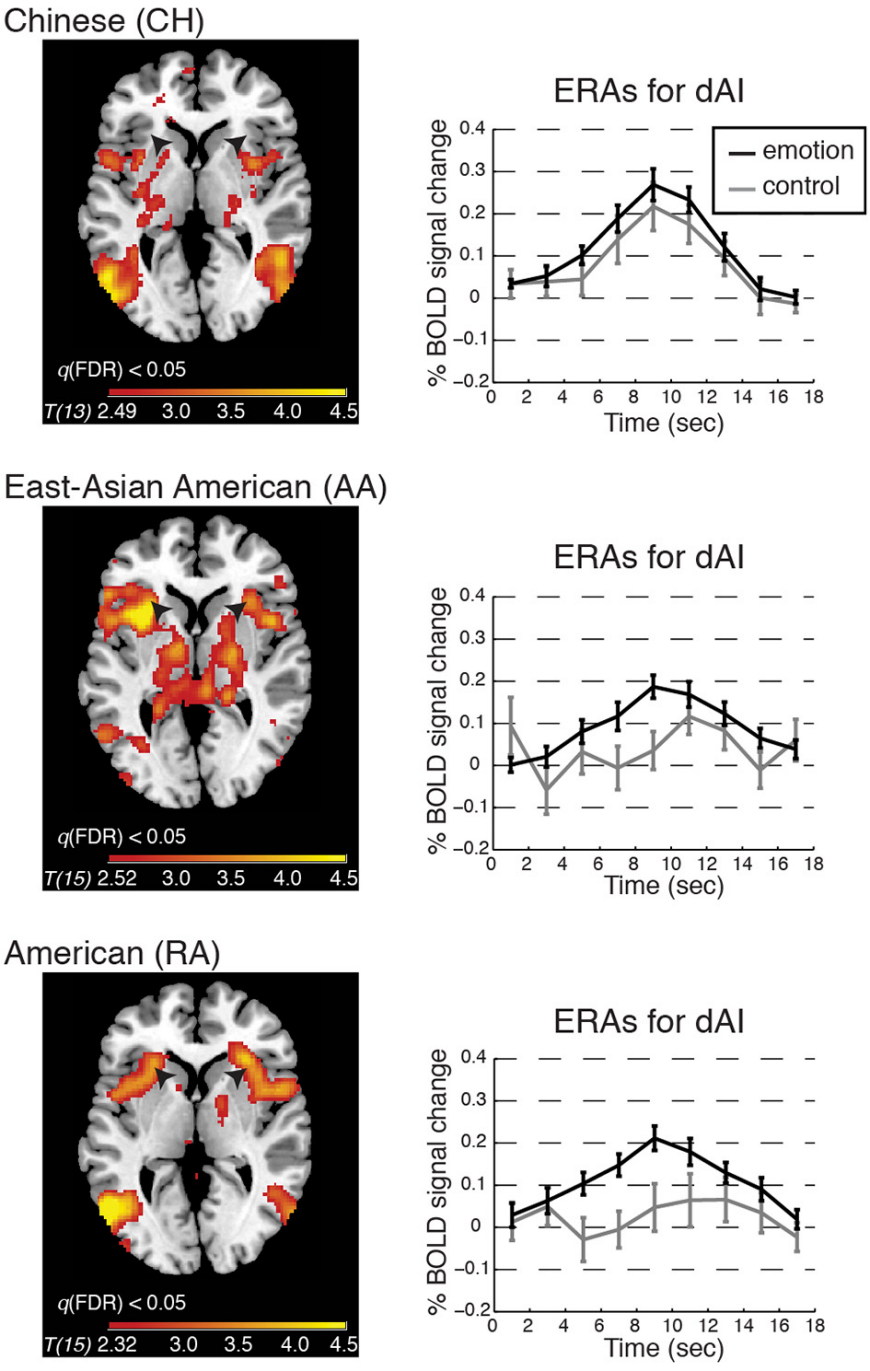
**BOLD contrasts and corresponding event-related averages (ERAs) of dorsal anterior insula (dAI) activity during emotion processing (in which emotion provoking narratives were shown and participants reported feeling emotional) and control processing (in which relatively unemotional narratives were shown and participants reported feeling no emotion)**. Contrasts are displayed on a template brain at *z* = 3 (MNI space), thresholded at *q*(FDR) < 0.05; arrows indicate the dAI. For simplicity we report the bilateral ERA results, as the left and right dAI showed no appreciable differences. ERAs are plotted with ±one standard error, uncorrected for hemodynamic delay. Notes: ERA BOLD responses for emotion reveal that all groups showed activation relative to baseline; however, only in the RA and AA groups was activation for emotion higher than for control. In the CH group, emotion and control resulted in comparable activation in the dAI (although other emotion-related regions showed significant activation for this contrast; see Table 2).

As expected, relative to baseline, each group showed significant activation in each AI VOI during emotion. Results in the dAI can be appreciated by viewing Figure 3, right hand panel. Activations of the vAI VOI (bilateral and averaged across the 4th–8th TR) were as follows: CH: *t*_[13]_ = 5.31, *p* < 0.001, Cohen's *d* = 1.42, 95% CI [0.10, 0.23]; AA: *t*_[15]_ = 4.72, *p* < 0.001, Cohen's *d* = 1.18, 95% CI [0.06, 0.16]; RA: *t*_[15]_ = 4.85, *p* < 0.001, Cohen's *d* = 1.21, 95% CI [0.07, 0.19].

#### Emotion vs. control processing

To begin to address whether BOLD activation reflected feeling strength, we examined each group's whole-brain BOLD activation pattern, and ERA plots, for emotion vs. control. We found that the dAI was activated for emotion relative to control in the RA and AA groups but not in the CH group; see Figure 3. The CH group showed comparable dAI activation for the emotion and control conditions. The vAI was activated for this contrast in all groups (see also Table 2).

**Table 2:** **BOLD maxima for emotion vs. control in the anterior insula and other emotion-related brain regions for Chinese and American participant groups, in MNI space, from separate whole-brain analyses**.

**Region**	**Chinese (CH)**				**East-Asian American (AA)**				**American (RA)**			
	**X**	**Y**	**Z**	***z* score**	**X**	**Y**	**Z**	***z* score**	**X**	**Y**	**Z**	***z* score**
Anterior insula												
Ventral	−44	8	−12	*3.64*	−38	−2	10	*3.31*	−46	8	−4	*3.37*
	42	6	−10	*3.91*	38	12	−12	*3.55*	48	12	−6	*4.33*
Dorsal					−32	12	4	*5.00*	−26	20	6	*4.05*
					32	22	4	*3.69*	30	22	10	*4.28*
3rd short gyrus/ principle sulcus	−38	6	8	*3.39*	−34	12	2	*4.91*	−46	8	0	*3.64*
	40	4	0	*3.13*	44	10	−2	*4.22*	42	8	12	*3.99*
Posterior insula	−36	−16	−4	*4.13*	−36	−8	12	*3.63*	42	−4	−10	*3.23*
Anterior cingulate	−2	−2	36	*4.2*	4	16	44	*3.35*	0	16	38	*4.84*
Posterior cingulate	−10	−26	36	*4.28*	−4	−34	34	*3.71*	−14	−38	44	*3.49*
	12	−24	48	*4.31*	8	−44	12	*3.43*	14	−36	44	*3.31*
Precuneus	−12	−56	62	*5.40*	−8	−74	50	*5.08*	−8	−60	62	*5.50*
	10	−52	64	*4.40*	2	−60	60	*3.97*	14	−66	52	*5.17*
Supramarginal gyrus	−60	−34	30	*3.94*	−58	−44	32	*3.34*	−56	−38	38	*6.42*
	68	−24	18	*3.11*	66	−38	28	*3.53*	62	−26	58	*5.36*
Mesencephalon					−12	−16	−8	*4.64*	−8	−24	−10	*4.04*
					12	−24	−12	*5.89*	12	−24	−12	*3.71*

*Activation thresholds were determined by the False Discovery Rate statistic, q(FDR) < 0.05. Note significant activation for emotion relative to control in the ventral anterior insula for all groups, but in the anterior-most portions of the dorsal anterior insula for the American groups only. (All groups showed activation in the dorsal sector of the 3rd short gyrus/principle sulcus of insula, in a location corresponding to the posterior border of the dorsal anterior insula. Voxels corresponding to this activation were included in the dAI VOI)*.

These results suggested that dAI activation was associated with feeling strength only in the American groups and that vAI activation was associated with feelings in all groups. We tested these possibilities and the relations of BOLD activity to interoceptive processing via the analyses presented below.

#### Trial-by-trial results

***Anterior insula***. As expected, even after controlling for feeling strength, BOLD magnitude was consistently positively correlated with cardiac arousal in each AI VOI (vAI: *t*_[45]_ = 3.83, *p* < 0.001, Cohen's *d* = 0.56, 95% CI [0.05, 0.15]; dAI: *t*_[45]_ = 4.87, *p* < 0.001, Cohen's *d* = 0.72, 95% CI [0.08, 0.19]). ANOVA revealed no effect of cultural group in either sector (vAI: *p* = 0.86, η^2^_*p*_ = 0.007; dAI: *p* = 0.87, η^2^_*p*_ = 0.006).

**Figure 4:**
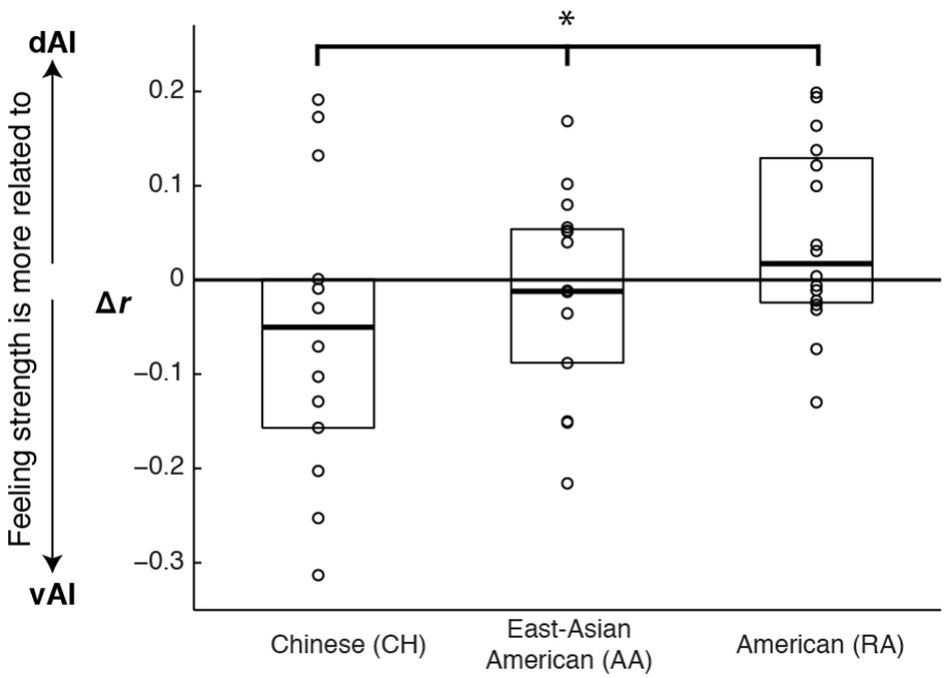
**Graph illustrating cultural differences in how ventral and dorsal anterior insula activation correlated with emotional feeling strength**. Scores represent the difference in the strength of correlation between dorsal and ventral activations and feelings (*r* dAI to feelings minus *r* vAI to feelings). Each data point represents one experiment participant. Boxes extend one quartile above and one quartile below the mean. The zero line represents equal correlation to feelings across the two AI sectors. A planned linear contrast revealed a significant difference across the cultural groups (^*^F_[1, 43]_ = 5.14, *p* = 0.028, η^2^_*p*_ = 0.107), and placed the AA group's results between those of the other groups.

As the results from the emotion vs. control contrasts suggested may be the case, controlling for cardiac arousal, the RA and CH groups showed a significant interaction between cultural group and AI sector on the relationship between BOLD and feeling strength (*F*_[1, 28]_ = 4.56, *p* = 0.042, η^2^_*p*_ = 0.140). A planned linear contrast placed the AA group's results between those of the CH and RA groups; see Figure 4.

To characterize the origins of this interaction, we examined each group's results. Controlling for cardiac arousal, we found that vAI BOLD magnitude was consistently positively correlated with feeling strength across participants in each of the cultural groups (CH: *t*_[13]_ = 4.23, *p* < 0.001, Cohen's *d* = 1.13, 95% CI [0.08, 0.24]; AA: *t*_[15]_ = 3.16, *p* = 0.006, Cohen's *d* = 0.79, 95% CI [0.04, 0.22]; RA: *t*_[15]_ = 2.96, *p* = 0.010, Cohen's *d* = 0.74, 95% CI [0.03, 0.17]). However, dAI magnitude was consistently correlated with feeling strength in the American groups only (CH: *t*_[13]_ = 1.75, *p* = 0.11, Cohen's *d* = 0.47, 95% CI [−0.02, 0.23]; AA: *t*_[15]_ = 2.67, *p* = 0.017, Cohen's *d* = 0.67, 95% CI [0.02, 0.21]; RA: *t*_[15]_ = 3.94, *p* = 0.001, Cohen's *d* = 0.98, 95% CI [0.07, 0.22]).

The cultural effect became especially pronounced when we examined the unique contributions of each AI sector to explaining feeling strength in each cultural group. Partialling out shared variance in BOLD magnitude between vAI and dAI, and controlling for magnitude of cardiac arousal, in the CH group vAI activation magnitude was consistently positively correlated with feeling strength but dAI activation magnitude was not (CH: vAI: *t*_[13]_ = 2.98, *p* = 0.010, Cohen's *d* = 0.80, 95% CI [0.03, 0.19]; dAI: *t*_[13]_ = −0.12, *p* = 0.91, Cohen's *d* = −0.03, 95% CI [−0.13, 0.12]). By contrast, in the RA group, vAI activation magnitude was not correlated with feeling strength, but dAI activation magnitude was (RA: vAI: *t*_[15]_ = −0.71, *p* = 0.49, Cohen's *d* = −0.18, 95% CI [−0.10, 0.05]; dAI: *t*_[15]_ = 2.95, *p* = 0.010, Cohen's *d* = 0.74, 95% CI [0.03, 0.19]). The AA group showed a shared variance pattern, intermediate between the CH and RA groups (AA: vAI: *t*_[15]_ = 1.27, *p* = 0.22, Cohen's *d* = 0.32, 95% CI [−0.04, 0.15]; dAI: *t*_[15]_ = 0.65, *p* = 0.53, Cohen's *d* = 0.16, 95% CI [−0.07, 0.12]).

***Posterior insula***. PI activity correlated robustly with cardiac arousal in each group (groups combined: *t*_[45]_ = 6.79, *p* < 0.001, Cohen's *d* = 1.00, 95% CI [0.13, 0.25]) even when controlling for vAI and dAI activity and feeling strength (groups combined: *t*_[45]_ = 5.33, *p* < 0.001, Cohen's *d* = 0.79, 95% CI [0.09, 0.19]). As expected, PI activity was not significantly correlated with feeling strength in any group (groups combined: *t*_[45]_ = 0.74, *p* = 0.47, Cohen's *d* = 0.11, 95% CI [−0.03, 0.07]). Controlling for PI activity, the pattern of correlations between vAI and dAI activity and feeling strength did not change from those reported above (i.e., all reported correlations were robust, and no new correlations emerged). This was true despite that controlling for PI activity rendered vAI and dAI correlations to cardiac arousal non-significant.

#### Post-hoc analysis of how dAI activation timing was related to decisions about feelings

The analyses above demonstrate that dAI activation magnitude was not related to feeling strength in the CH group. However, they leave open the question of whether the dAI activation observed across groups relative to baseline was related to a cognitive process that is invoked for decisions about feelings. In the CH group, this process could have been invoked no matter the feeling strength. Supporting this interpretation, we found that in all groups the timing of the dAI peak BOLD activation tracked the time participants required to decide how strongly they felt (i.e., positively correlated with button press response time; CH: *t*_[13]_ = 3.80, *p* = 0.002, Cohen's *d* = 1.02, 95% CI [0.08, 0.28]; AA: *t*_[15]_ = 3.09, *p* = 0.007, Cohen's *d* = 0.77, 95% CI [0.04, 0.21]; RA: *t*_[15]_ = 2.43, *p* = 0.028, Cohen's *d* = 0.61, 95% CI [0.02, 0.26]). This effect was not seen in the ventral sector (CH: *t*_[13]_ = 1.15, *p* = 0.27, Cohen's *d* = 0.31, 95% CI [−0.04, 0.12]; AA: *t*_[15]_ = 0.67, *p* = 0.51, Cohen's *d* = 0.17, 95% CI [−0.07, 0.14]; RA: *t*_[15]_ = 1.33, *p* = 0.20, Cohen's *d* = 0.33, 95% CI [−0.03, 0.12]). We also note that button-pressing was right handed but that the correlation between dAI activation timing and response time held bilaterally, making it unlikely to be attributable to the motor act of pressing the response button.

#### Replication

**Figure 5:**
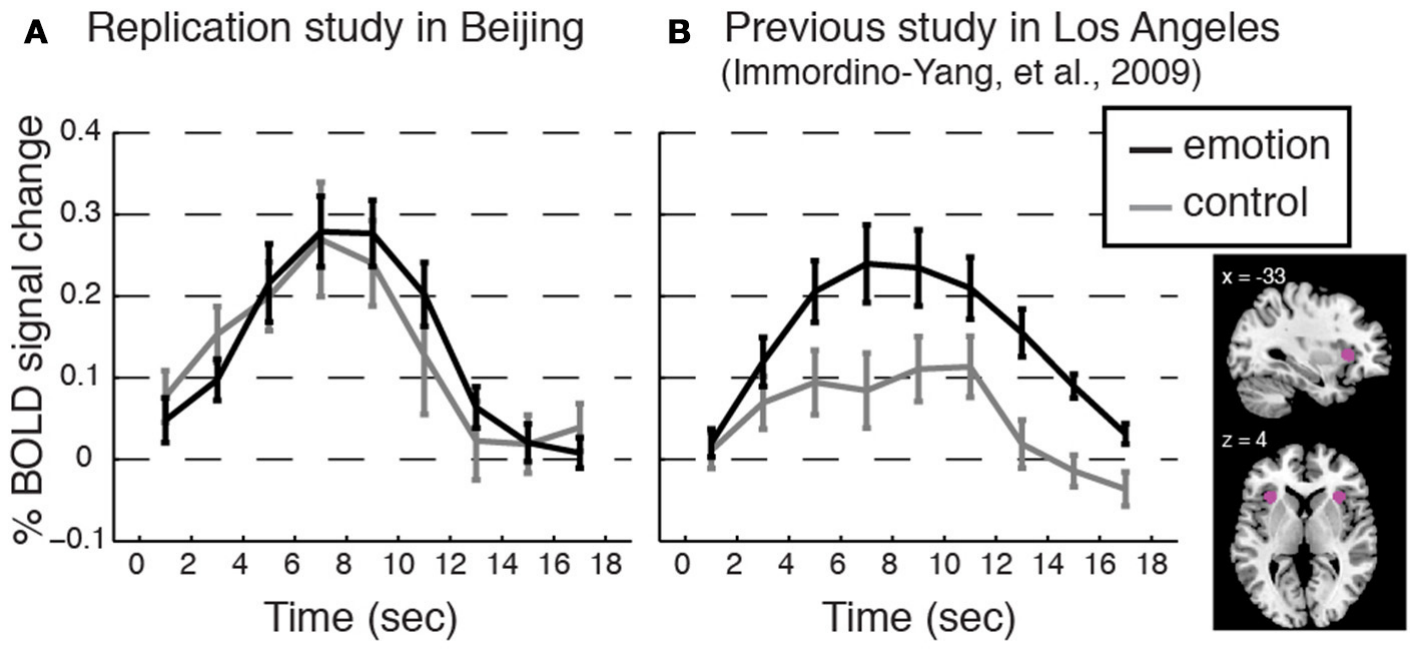
**Replicating the cultural group difference in how dAI activation correlated with participants' experienced feeling strength**. Event-related averages for the dorsal anterior insula (dAI) BOLD response time courses for social emotion and control from **(A)** an additional study conducted with a separate group of Beijing Normal University students (*n* = 14; 7 females; average age 22.8, *SD* = 2.43; monolingual Mandarin-speaking) and from **(B)** a previous study with American participants (Immordino-Yang et al., 2009; *n* = 13; participants were native English-speaking). Plots are as in Figure 3. Methods: Voxels were taken from 6 mm spheres centered at *x* = ± 33; *y* = 22; *z* = 4, depicted in pink in the inset. The protocol, scanner model, functional scanning sequence, and data analysis techniques were equivalent between these two studies and the current study except that ECG was not collected, and the narrative stimuli were from a different corpus than those used in the current study. Protocol Validation: All participants reported feeling emotional during the experiment, and there were no differences in the strength of feelings that Chinese participants reported over the course of the experiment as compared to the strength of feelings the American participants reported, *t*_[24]_ = 1.33, *p* = 0.20, Cohen's *d* = 0.39, 95% CI [−0.70, 0.15]. Results: Consistent with the main study, the dAI is comparably activated for both stimulus types in the data from Chinese participants, while the dAI shows stronger activation for emotion than for control processing in the data from American participants. Trial-by-trial analyses reinforced this result: Feeling strength was not correlated with dAI activation level trial-by-trial in the Chinese replication group: *t*_[13]_ = 1.15, *p* = 0.27, Cohen's *d* = 0.31, 95% CI [−0.05, 0.16]; but, feeling strength was correlated with dAI activation in the American group: *t*_[11]_ = 4.38, *p* = 0.001, Cohen's *d* = 1.26, 95% CI [0.08, 0.25]. (Button press values were missing for one American participant.)

We successfully replicated the cultural group difference in the correspondence between dAI activation and feeling strength using data previously collected for two studies of the same social emotions, one involving 13 American participants in Los Angeles (i.e., a novel analysis of the data first presented in Immordino-Yang et al., 2009) and one involving 14 Chinese participants in Beijing (not previously published; see Figure 5).

## Discussion

### Cultural group differences in the neural processing of social-emotional experiences

We interpret our findings first to suggest that the neural process by which social emotions are experienced is relatively independent from interoception at the cortical level. In three separate participant groups, one Chinese and two American, cardiac arousal and participants' reports of emotional feeling strength contributed independently to explaining variance in AI activity as participants reacted to emotion inducing narratives. In addition, controlling for PI activity rendered AI correlations to cardiac arousal negligible and non-significant, but did not alter patterns of correlation between AI activity and feelings. These findings are somewhat unexpected given current neurobiological accounts of feelings. However, they accord well with psychological accounts that emotional feelings are conceptually mediated and reinforce the possibility that this conceptual mediation is acquired from social encounters and shaped by cultural norms.

**Figure 6:**
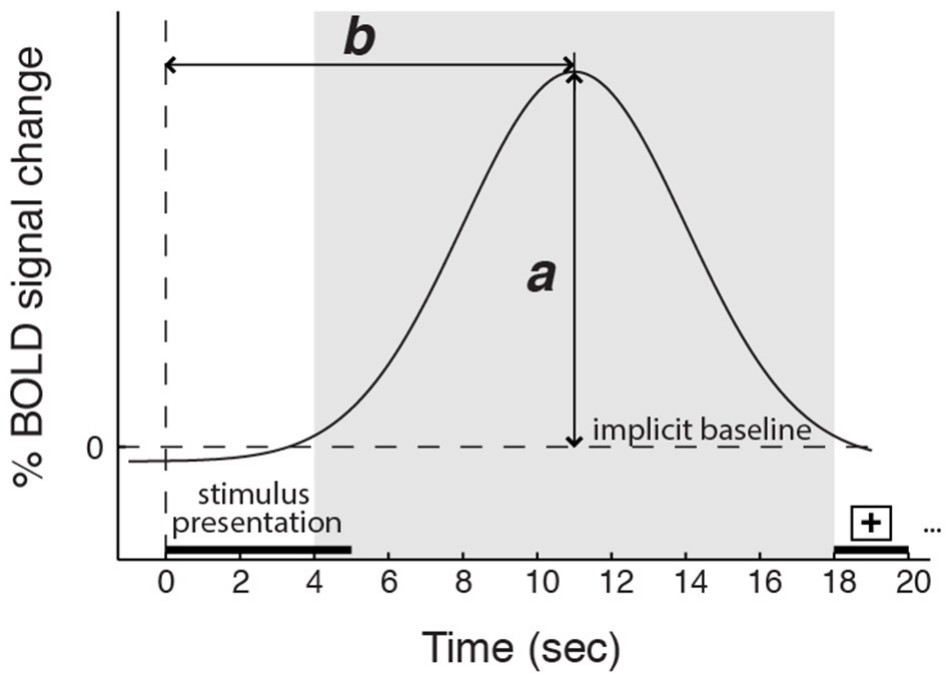
**Schematic depicting the magnitude (*a*) and the timing (*b*) of the peak BOLD response**. The shaded region represents the time window in which the BOLD signal peak was identified. Five-second video stimuli were shown starting at time 0, followed by 13 s of gray screen and 2 s of a fixation cross. Controlling for cardiac arousal: for the dorsal anterior insula, ***a*** is related to feeling strength, but only in the American participant groups; ***b*** is related to the time participants required to report a decision about their feeling strength in all groups. For the ventral anterior insula, ***a*** is related to feeling strength for all groups; ***b*** is unrelated to reports of feeling strength.

Aligned with this possibility, we also found group differences in how variance in AI activity correlated with participants' reports of their experienced feeling strength. Most notably, in the data from three American participant groups (two collected for the main study and one collected for a previous study, Immordino-Yang et al., 2009), dAI activation was greater the more strongly emotional participants reported feeling. By contrast, in the data from two Chinese participant groups (one collected for the main study and one utilized to replicate the main finding), dAI activation magnitude was unrelated to the strength of social-emotional feelings participants reported experiencing. This was true despite an absence of cultural group differences in the magnitude of AI BOLD signal change to emotion stimuli and an absence of group differences in participants' reported strength of feelings across the experiment. Notably, we did find that the magnitude of activity in the vAI correlated with feeling strength across the groups in the main study, and that the timing of the peak activation in the dAI was specifically related to the time at which individuals reported their emotional feeling (see also Figure 6).

Taking these findings together, dAI activity in the CH group appears to have reflected decision timing but not feeling strength, while dAI activity in the RA and AA groups appears to have reflected both decision timing and feeling strength. We interpret this difference as evidence that culture may influence the process by which individuals construct conscious experiences of social emotion, even though, as expected, we found no evidence that culture influences basic interoceptive processing. The broad psychological implication is that conscious assessment of emotion strength is subjective and context dependent—in essence, it appears to reflect cultural strategies that are deployed relatively independently of the body's emotion-related visceral changes (see sections Implications for the Psychological Process by Which Emotions are Experienced and Arousal vs. Feeling Strength, for additional interpretation).

### Ventral and dorsal AI contributions to emotional feelings

Our findings are relevant to open questions concerning how the insula supports feelings. In particular, research on somatic stimulation has demonstrated that PI activity increases proportionately to actual body stimulation (such as proportionately to temperature increases in a thermal stimulus) but AI activity increases proportionately to an individual's affective, context-dependent experience of the somatosensory stimulus (e.g., as subjectively painful or not; Craig et al., 2000). Our findings are consistent with assertions that it is therefore the AI that supports conscious feelings, including feelings of emotion (Craig, 2002).

Our findings are also consistent with the known cognitive functions of the dAI (e.g., Mutschler et al., 2009; Kurth et al., 2010; Nelson et al., 2010; Touroutoglou et al., 2012), with suggestions that the dAI supports emotion awareness (Craig, 2002; Critchley et al., 2004; Gu et al., 2013), and with assertions that the vAI's functioning is related to strength of subjective affect, or feelings (e.g., Kurth et al., 2010; Touroutoglou et al., 2012). However, our findings suggest that the ventral and dorsal AI sectors' relative contributions to feelings are influenced by exposure to culture: in the CH group, feeling strength was more associated with vAI activation, while in the RA group, feeling strength was more associated with dAI activation. The intermediate results from the AA group, which is bicultural, support the interpretation that the CH to RA group difference is related to cultural exposure.

Considering the complexity of the AI's anatomical and functional connectivity (Mesulam and Mufson, 1982b; Mufson and Mesulam, 1982; Seeley et al., 2007; Menon and Uddin, 2010; Nelson et al., 2010) and the fact that this region's connectivity changes across child development (Uddin et al., 2011), the cultural differences we found could be said to open more questions than they answer. Especially given the pivotal role the AI plays in functional network activation (Menon and Uddin, 2010), it is unlikely that the cultural effects we observed are limited to this region or to the processing of social-emotional feelings. The AI is involved in a variety of basic processes such as attention orienting (Menon and Uddin, 2010; Nelson et al., 2010), chemosensory perception (e.g., for taste and olfaction, Mesulam and Mufson, 1982b; Small, 2010) and experiencing and observing others experiencing disgust (Wicker et al., 2003). It is also involved in more complex psychological processes such as affective processing and regulation (Craig, 2002; Wager and Barrett, 2004; Paulus and Stein, 2006), decision-making (Brass and Haggard, 2010; Naqvi and Bechara, 2010; Naqvi et al., 2014), moral judgment (Moll et al., 2005), self-referential processing (D'Argembeau et al., 2012), and social emotions such as sympathy (Decety and Michalska, 2010), empathy (Singer et al., 2004), rejection (Eisenberger et al., 2003; Cacioppo et al., 2013), compassion (Bruneau et al., 2012), and love (Bartels and Zeki, 2004; Cacioppo et al., 2012). Our findings point toward the need to investigate possible cultural differences in AI activity and network connectivity across various domains of processing, as well as the need for studies probing the developmental mechanisms by which culture or experience (e.g., with compassion meditation; Lutz et al., 2009) may be organizing or biasing this region's functioning.

In future investigations, it will be important to keep in mind that, despite ubiquitous assumptions to the contrary, similar levels/locations of neural activity across individuals or groups of participants during an experimental task may not correspond to similar psychological processing. In our study, the cultural group differences were found in the patterns of correlation between BOLD signal magnitude and participants' reports of their experienced feeling strength, rather than in the magnitude of the BOLD signal itself. This result underscores the need to include relevant psychological measures in future investigations of possible cultural and individual similarities and differences in activity, and in anatomical and functional connectivity, of the insula and other neural systems (Immordino-Yang, 2013).

### Implications for the psychological process by which emotions are experienced

One potential explanation for our findings is that individuals from more expressive cultures, like American culture, learn over time to rely more heavily on somatosensory processing mechanisms in deciding their strength of feelings, as there would presumably be more information in the body response (or simulated, predicted or “as-if” body response; Damasio, 1999; Seth, 2013). In more expressive individuals, feelings could therefore become more closely associated with the activity of the dAI because of its somatosensory properties. By contrast, cultural groups that value calmness, like Chinese, may learn over time that mechanisms of autonomic modulation provide more important clues to emotion strength; in these groups, conscious feelings may become more associated with the activity of the vAI because of its modulatory role. These developmental mechanisms could hold whether or not different categories of emotion are associated with distinct or culturally universal patterns of behavior (cf. Jack et al., 2012; Nummenmaa et al., 2014), and whether or not emotions are found to have categorically distinct neural correlates (Hamann, 2012). This proposed mechanism would also be consistent with evidence that European Americans have a stronger ability to become consciously aware of heart-beat sensations than do Asian Americans on average, and that this ability has been associated with individuals' likelihood of experiencing non-socially induced emotional arousal as relevant to the social context (Ma-Kellams et al., 2012).

In support of this idea, in a separate study (Immordino-Yang et al., under review) we demonstrated that approximately 15% of the inter-individual variability in the correspondence between dAI activations and feeling strength across the groups is explained by participants' natural emotional expressiveness in a private interview outside of the MRI scanner. It would be interesting in the future to investigate additional psychological, behavioral, and neural correlates of the considerable inter-individual variability in the correspondence between BOLD activity and feeling strength to determine whether it may relate to other known sources of individual and cultural variation in emotion-related values, experiences and behavior (Ekman, 1971; Markus and Kitayama, 1991; Mesquita and Frijda, 1992; Eid and Diener, 2001; Tsai, 2007; Matsumoto et al., 2008) and neural activity (Han and Northoff, 2008; Cheon et al., 2013; Chiao et al., 2013; Han et al., 2013). Such studies could begin by investigating how the effects we discovered could be relevant to understanding variability in the role of bodily reactions in the experience of emotion (Tsai et al., 2004; Barrett et al., 2007; Chentsova-Dutton and Tsai, 2010; Dunn et al., 2010; Ma-Kellams et al., 2012), and to mental and physical health, for example to understanding cultural differences in the prevalence of somatization disorders (Parker et al., 2001; Ryder et al., 2008) and cultural and social contextual effects on pain experience and tolerance (e.g., Edwards et al., 2001; Eisenberger et al., 2006; Rahim-Williams et al., 2007).

Overall, our findings lend credence to efforts to study cultural and individual variability in the construction of emotional feelings, and to efforts to understand how this variability may contribute social-emotional liabilities or sources of resilience depending on individuals' predispositions and on the context. A highly productive literature has linked the effects of suboptimal social environments and social relationships, such as are associated with poverty, abuse and neglect, to brain development and maladaptive socio-emotional functioning (Cicchetti, 2004; Hackman et al., 2010). Our study suggests that exposure to culture, which is a normative, positive source of adaptive variability in social development, also influences how individuals experience emotions.

### Arousal vs. feeling strength

Importantly, it is unlikely that the cultural difference we found in the neural processing of feelings is attributable to interoceptive processing of arousal. Although of course the AI maps visceral states beyond those associated with the heart, changes in cardiac activity are arguably among the most robust and salient visceral manifestations of emotional arousal. Yet, the cultural effect we found held after controlling for variance in AI activity that correlated with cardiac arousal and after controlling for PI activity, which rendered AI correlations to cardiac arousal non-significant and negligible. A follow-up analysis revealed that feeling strength and cardiac arousal were not significantly correlated trial-by-trial in any group (groups combined: *t*_[45]_ = 1.29, *p* = 0.20, Cohen's *d* = 0.19, 95% CI [−0.02, 0.10]). Our findings therefore accord well with psychological evidence that experiences of emotion—conscious feelings—do not correspond in one-to-one fashion with measures of body arousal or with embodied sensations (Barrett et al., 2007). They also accord with reports that contributions of arousal and body sensations to emotional feelings and to emotion-related somatosensory neural activations vary across people and contexts (Barrett et al., 2007; Dunn et al., 2010; Saxbe et al., 2013).

### Notes concerning the particular emotions our stimuli induced

Although this was not the main focus of our study, it is notable that our findings appear to be consistent across the range of positively and negatively valenced social emotions we induced (see also Supplementary Figure 1). Some of our stimuli (meant to induce varieties of admiration) induced rewarding, energizing, positive feelings, often described by participants as feelings of inspiration, motivation, and amazement; others of our stimuli (meant to induce varieties of compassion) induced painful feelings, and participants often described feeling “awful” or “bad.” This difference does not seem to have been reflected in our findings.

In addition, the feelings we tested varied on the extent to which they require complex social-cognitive inferences about the protagonist's broader situation, or instead can be more directly, empathically induced by mirroring the emotion that the protagonist shows. Emotions like admiration for virtue and compassion for social pain require complex inferences about the protagonist's experience relative to his or her cumulative life circumstances. By contrast, reactions to another's painful injury are relatively automatic and empathic. Here, the participant vicariously experiences the protagonist's physical pain and need not reflect much on the person's character traits or personal history to appreciate the protagonist's current emotion. Future studies will need to address these issues more fully, and to test whether our results extend beyond the prosocial, affiliative emotions we studied to antisocial emotions like hate, moral disgust, or contempt, to empathic emotions other than compassion for physical pain (like empathic happiness or pride), to varieties of non-social emotions like disgust to sanitation hazards, or to feelings unrelated to emotion, like hunger or physical pain.

## Conclusion

In sum, we found that AI processing of cardiac arousal, a basic function developmentally and evolutionarily, was consistent across cultural groups, and that AI processing of feelings, a highly developed psychological capability, showed cultural effects. Although this study does not address the origins of these cultural effects, the findings suggest that the ability of the human brain to construct conscious feelings of social emotion is less closely tied to visceral states than many neurobiological models predict and at least partly culturally acquired.

## Author contributions

Mary Helen Immordino-Yang and Hanna Damasio initiated a cross-cultural experiment. Mary Helen Immordino-Yang and Xiao-Fei Yang designed the experiment, collected and analyzed the data. Mary Helen Immordino-Yang and Xiao-Fei Yang wrote the paper, and Hanna Damasio provided critical feedback.

### Conflict of interest statement

The authors declare that the research was conducted in the absence of any commercial or financial relationships that could be construed as a potential conflict of interest.
