# Supplementary material for: Correlations between social-emotional feelings and anterior insula activity are independent from visceral states but influenced by culture
Source: Front Hum Neurosci. 2014 Sep 16;8:728. doi: 10.3389/fnhum.2014.00728 (PMC4165215; doi:10.3389/fnhum.2014.00728)
Supplement: Supplementary file 1 [file DataSheet1.PDF]

Supplementary Material

Correlations between social-emotional feelings and anterior insula activity are independent from visceral states but influenced by culture

Mary Helen Immordino-Yang,[1,2,3]* Xiao-Fei Yang,[1,3] Hanna Damasio[1,3,4]

[1] Brain and Creativity Institute, [2] Rossier School of Education,
[3] Neuroscience Graduate Program, [4]Dornsife Cognitive Neuroscience Imaging Center,
University of Southern California, Los Angeles, CA 90089, USA.

*Correspondence to: Mary Helen Immordino-Yang, 3620A McClintock Ave, Room 267, Los Angeles, CA 90089-2921
immordin@usc.edu

1. Supplementary Analyses

1.1 Testing for effects of in-group versus out-group narrative protagonist on anterior insula BOLD response

We tested whether there was an effect of an interaction between the participants' cultural group and the narrative protagonists' nationality on the magnitude of the BOLD response in the insula VOIs. Using the method described in the main paper, we calculated for each individual an ERA of the BOLD data from all trials in which a Chinese protagonist was shown, and another from all trials in which an American protagonist was shown. For each ERA, we averaged the BOLD signal magnitude of the 4th-8th TR's, and the resulting values were entered into a repeated measures ANOVA using protagonists' nationality as a within-subject factor and participants' cultural group as a between-subject factor. We found no effect of an interaction between the participants' cultural group and the nationality of the stimulus protagonist on the BOLD magnitude in the AI VOIs (vAI: $F[2,43] = 1.42$, $p = 0.25$, $\eta_p^2 = 0.062$; dAI: $F[2,43] = 1.14$, $p = 0.33$, $\eta_p^2 = 0.050$) .

1.2 Testing for effects of in-group versus out-group narrative protagonist on feeling strength

We tested whether there was an effect of an interaction between the participants' cultural group and the narrative protagonists' nationality on participants' reported feeling strength. In a repeated measures ANOVA, participants' average feeling strength ratings to all narratives depicting Chinese and American protagonists were entered as within-subject factors and participants' cultural group was entered as a between-subject factor. We found no effect of an interaction between the participants' cultural group and the

nationality of the stimulus protagonist on the strength of feelings participants reported ($F[2,43] = 0.456$, $p = 0.64$, $\eta_p^2 = 0.020$).

2. Supplementary Tables and Figures

2.1 Supplementary Tables

Supplementary Table 1. *T* statistics, corresponding *p*-values and 95% confidence intervals for BOLD activation relative to implicit baseline in dAI and vAI VOIs for trials in which positive stories were shown (inducing admiration) and trials in which negative stories were shown (inducing compassion), by cultural group.

	**Chinese (CH)** ($df = 13$)		**East-Asian American (AA)** ($df = 15$)		**American (RA)** ($df = 15$)	
	**dAI**	**vAI**	**dAI**	**vAI**	**dAI**	**vAI**
**Positive**	$t = 5.26$, $p < 0.001$ [0.09, 0.22]	$t = 5.04$, $p < 0.001$ [0.09,0.23]	$t = 4.03$, $p = 0.001$ [0.05, 0.16]	$t = 2.89$, $p = 0.011$ [0.02, 0.15]	$t = 7.38$, $p < 0.001$ [0.10, 0.18]	$t = 4.19$, $p = 0.010$ [0.07, 0.21]
**Negative**	$t = 6.25$, $p < 0.001$ [0.11, 0.24]	$t = 5.11$, $p < 0.001$ [0.10, 0.24]	$t = 5.67$, $p < 0.001$ [0.10, 0.22]	$t = 4.55$, $p < 0.001$ [0.07, 0.19]	$t = 7.07$, $p < 0.001$ [0.11, 0.21]	$t = 4.38$, $p < 0.001$ [0.06, 0.18]

2.2 Supplementary Figures

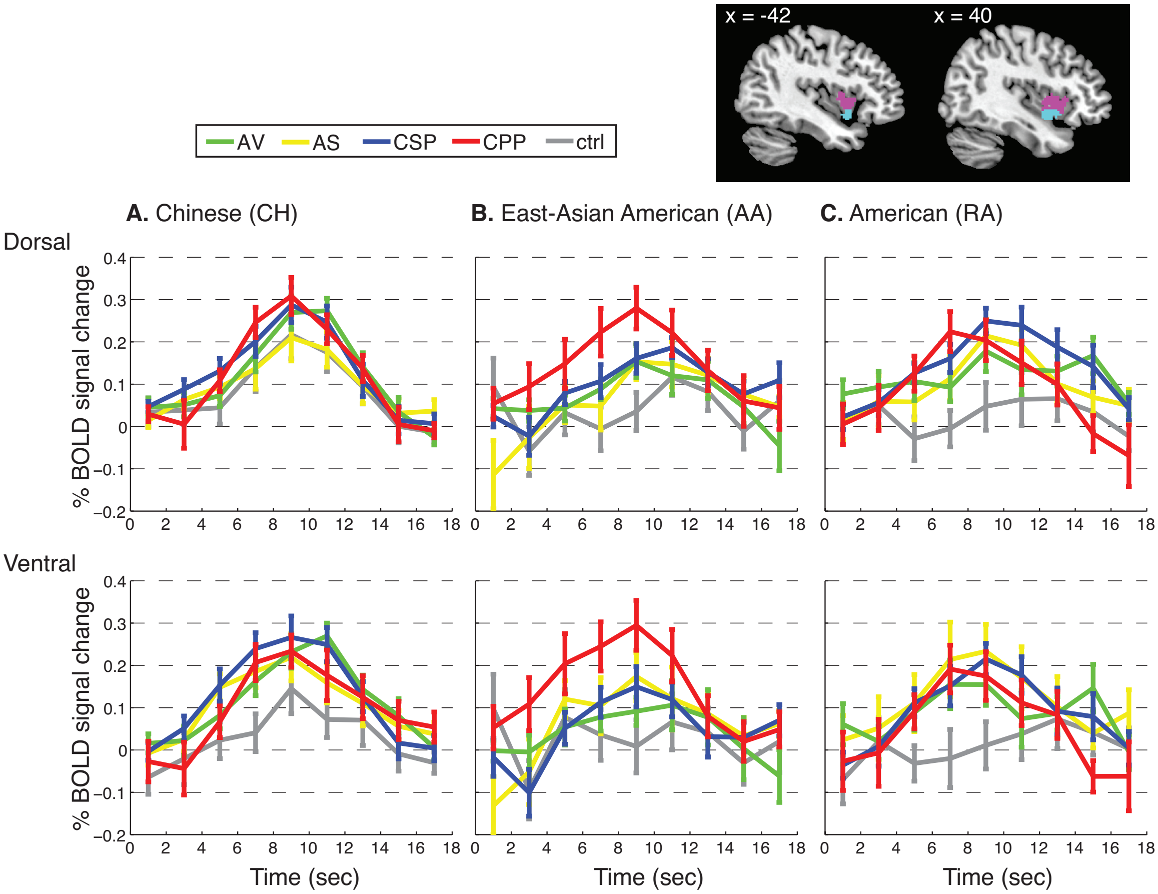

Supplementary Figure 1. Event-related averages for the dAI and vAI VOI BOLD response time courses for each sub-category of emotion stimuli (separately plotted, in colors) and control stimuli (in gray) for the three participant groups, with +/- one standard error, uncorrected for hemodynamic delay. Only emotion trials in which participants reported feeling emotional, and control trials in which participants reported feeling unemotional, are included. The VOIs are as in (main) Figure 1. Stimulus subcategories: admiration for virtue (AV, green; positive stories of remarkable altruism were shown); admiration for skill (AS, yellow; positive stories depicting skillful feats were shown); compassion for social pain (CSP, blue; negative stories of social exclusion or grief were shown); compassion for physical pain (CPP, red; negative stories in which individuals sustain accidental physical injuries were shown). Relatively less emotional social control stories are plotted in gray (ctrl). **NOTE:** All plots show higher activation for emotion than for control processing except for the dAI in the Chinese group.
